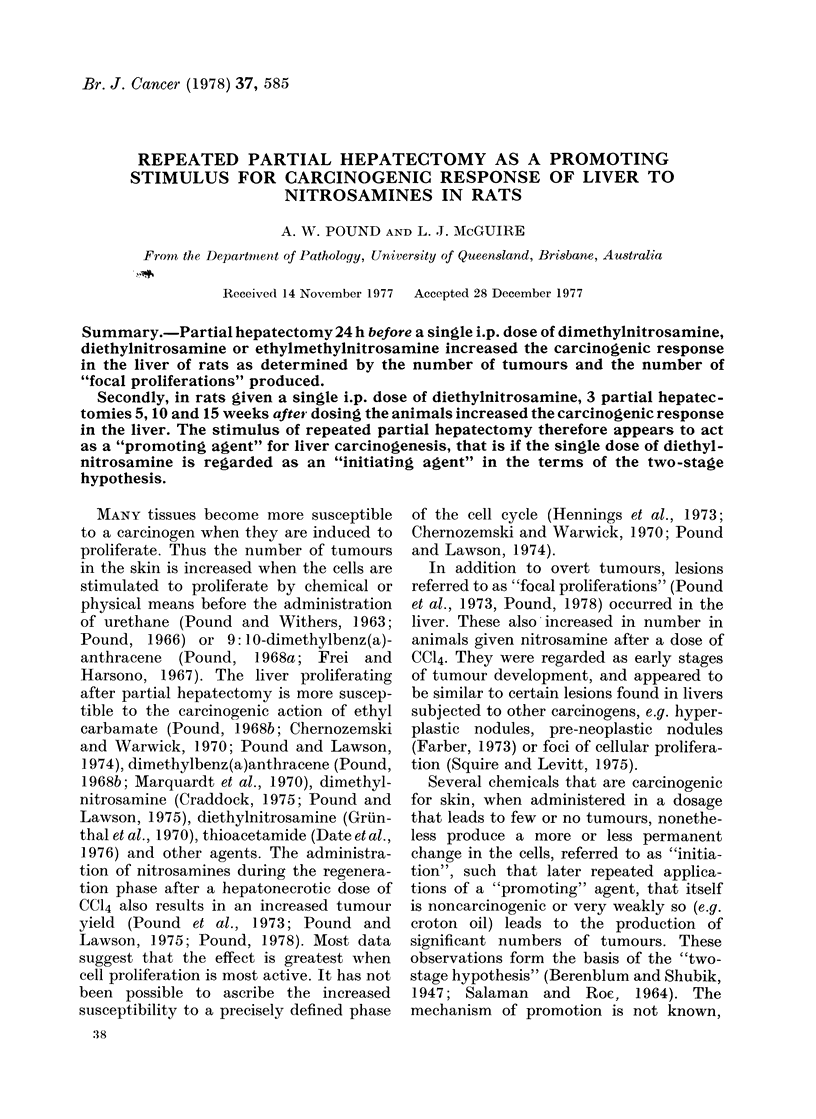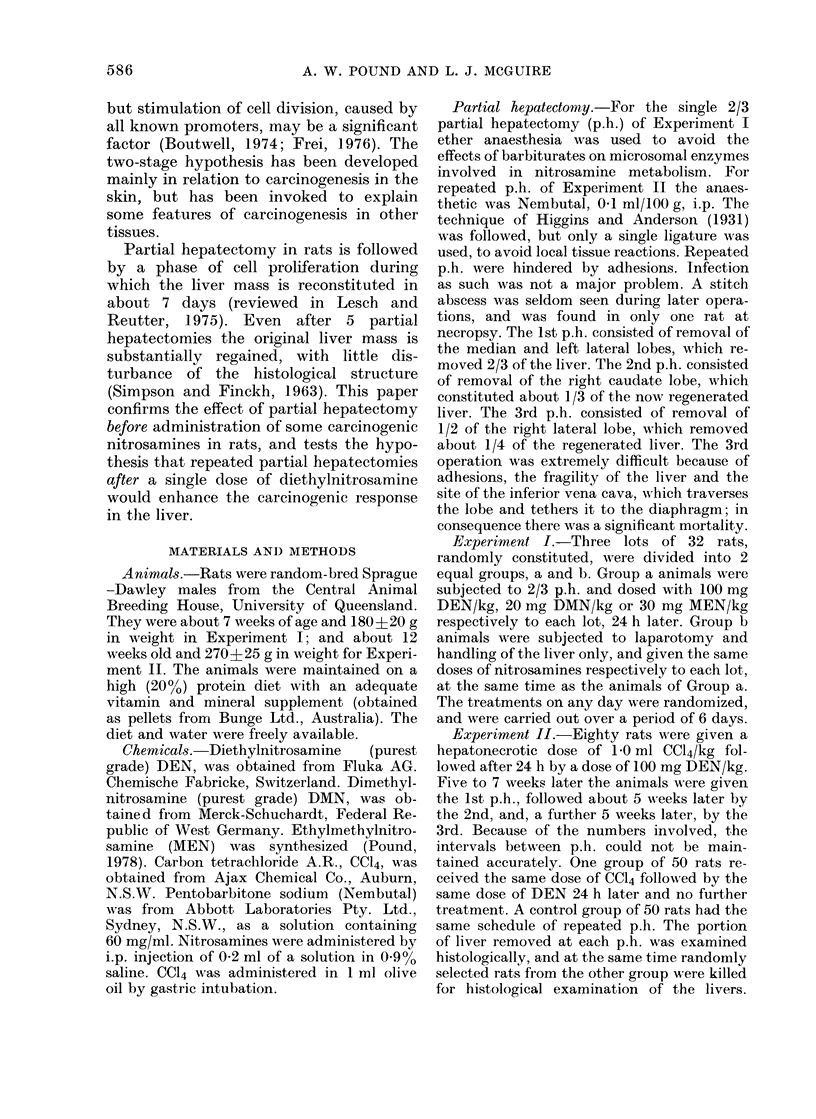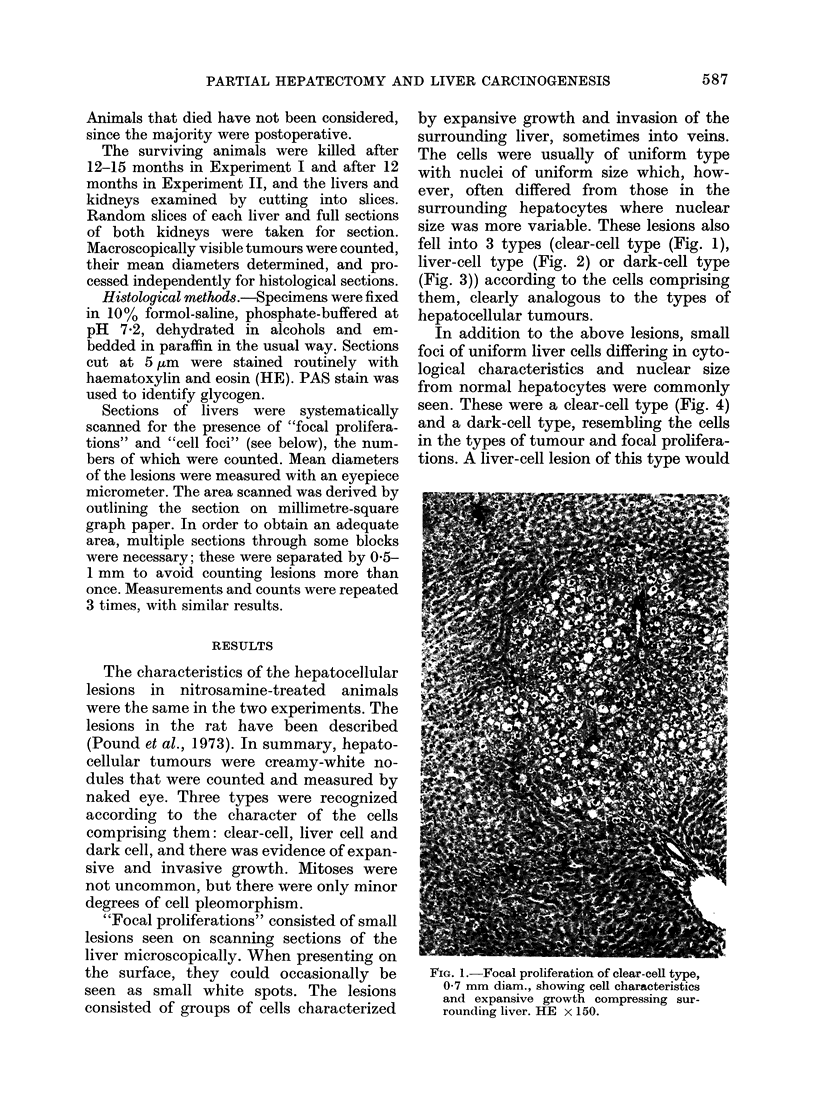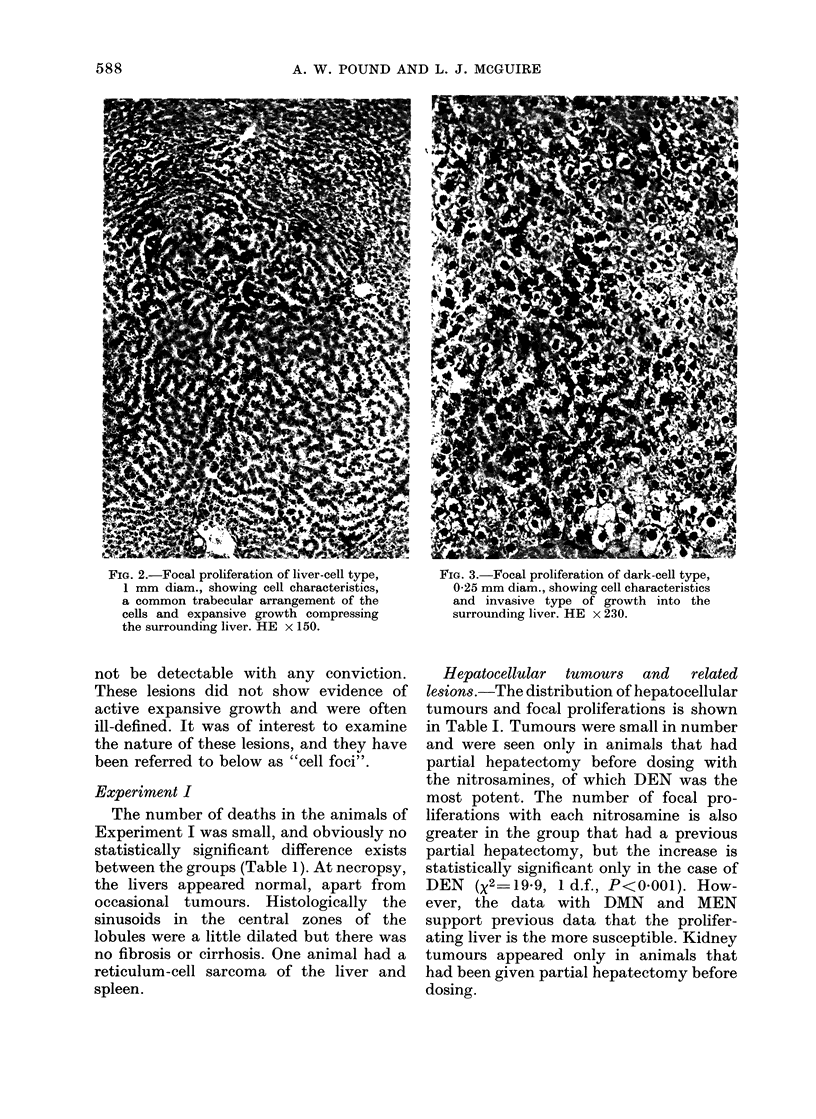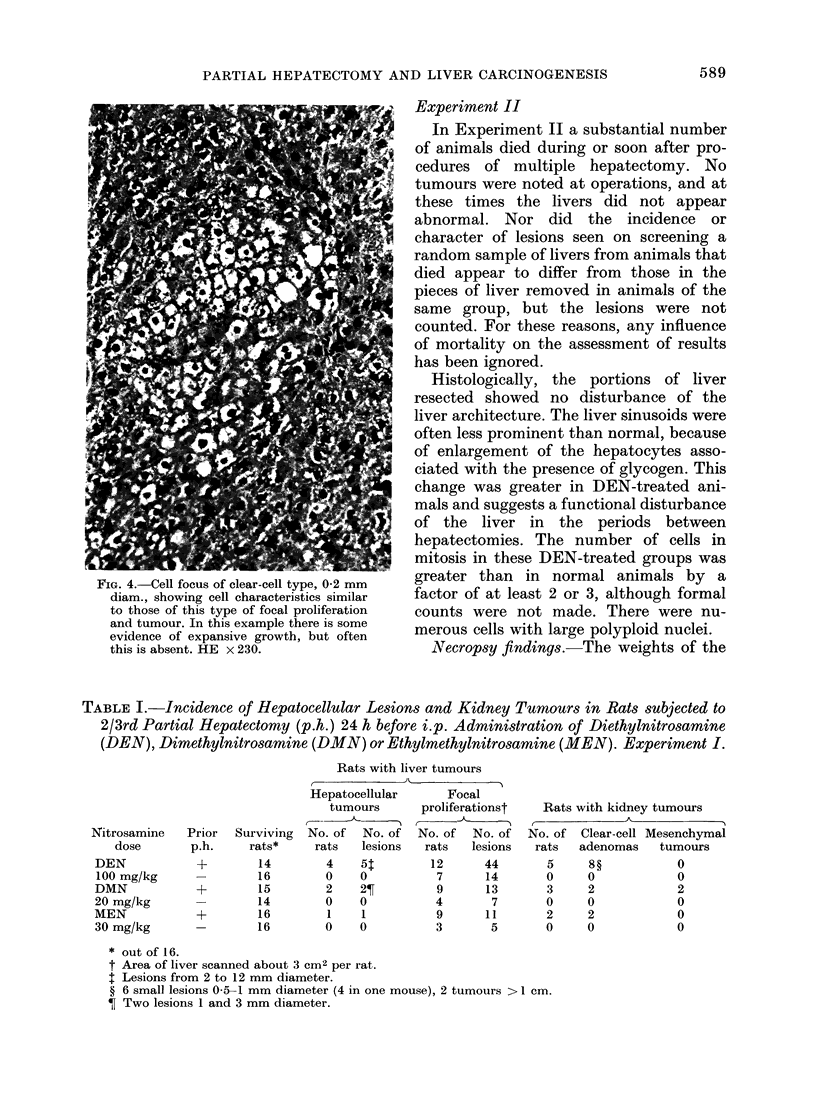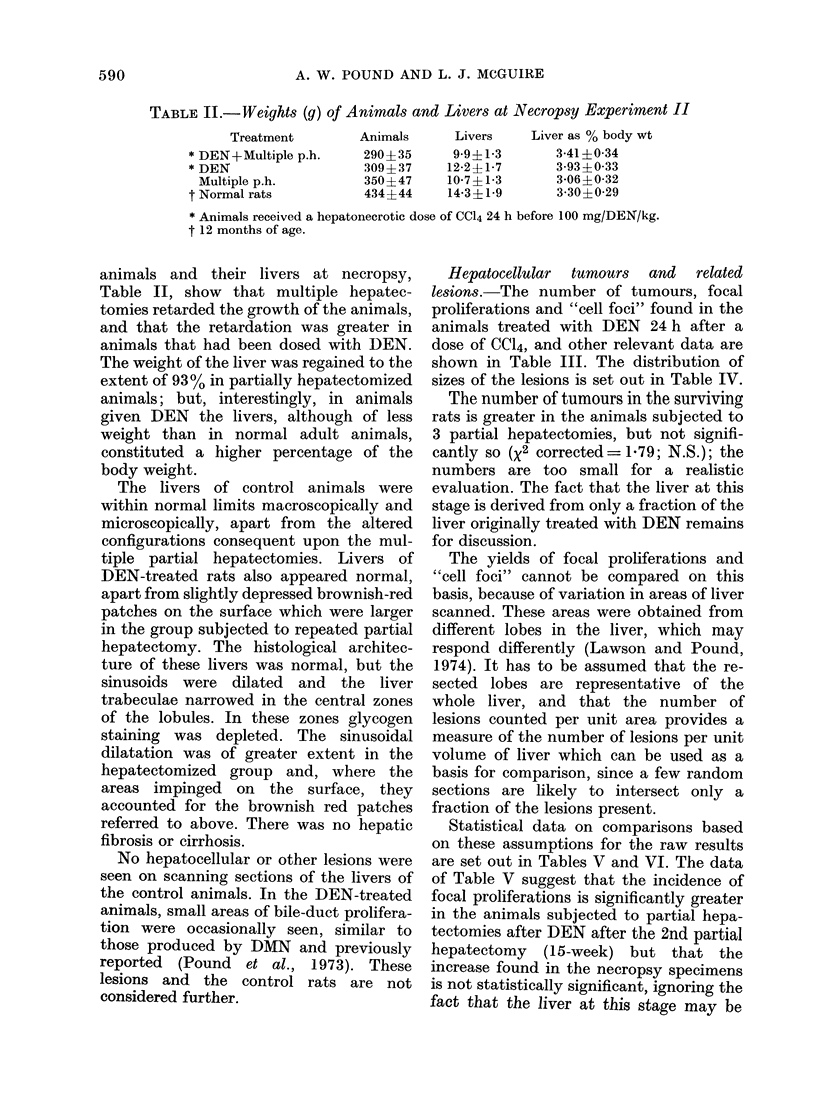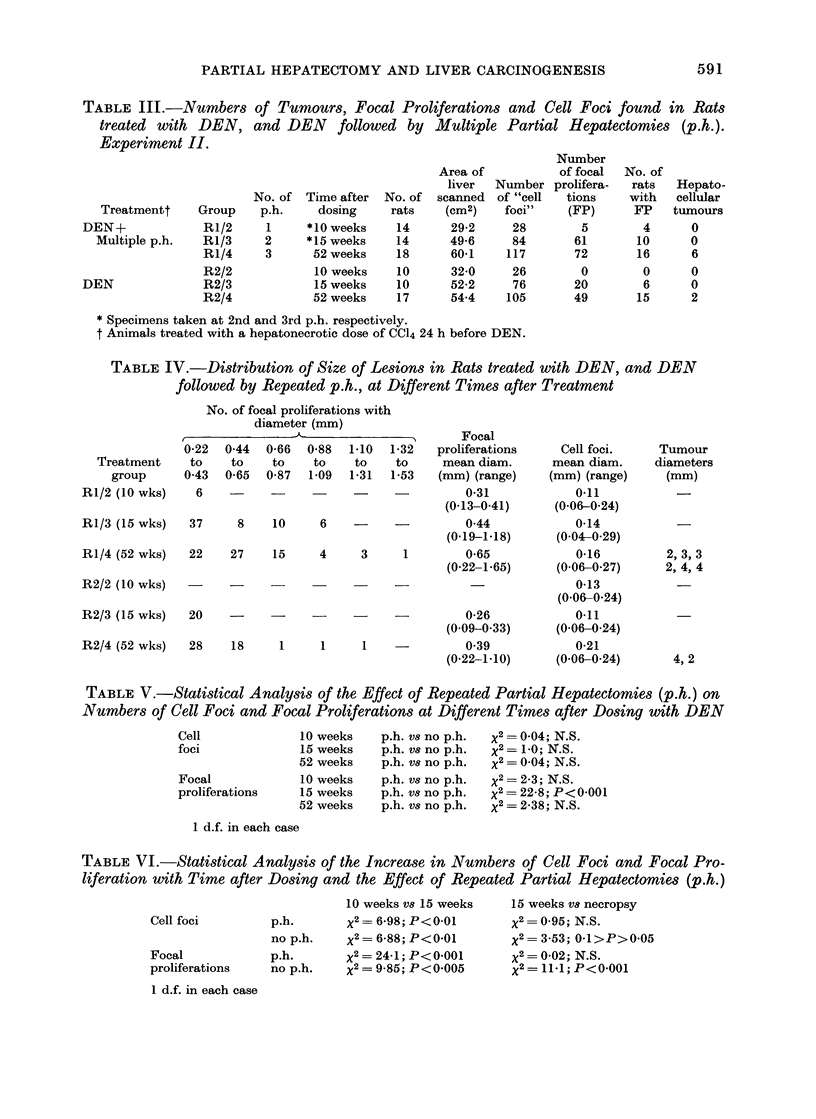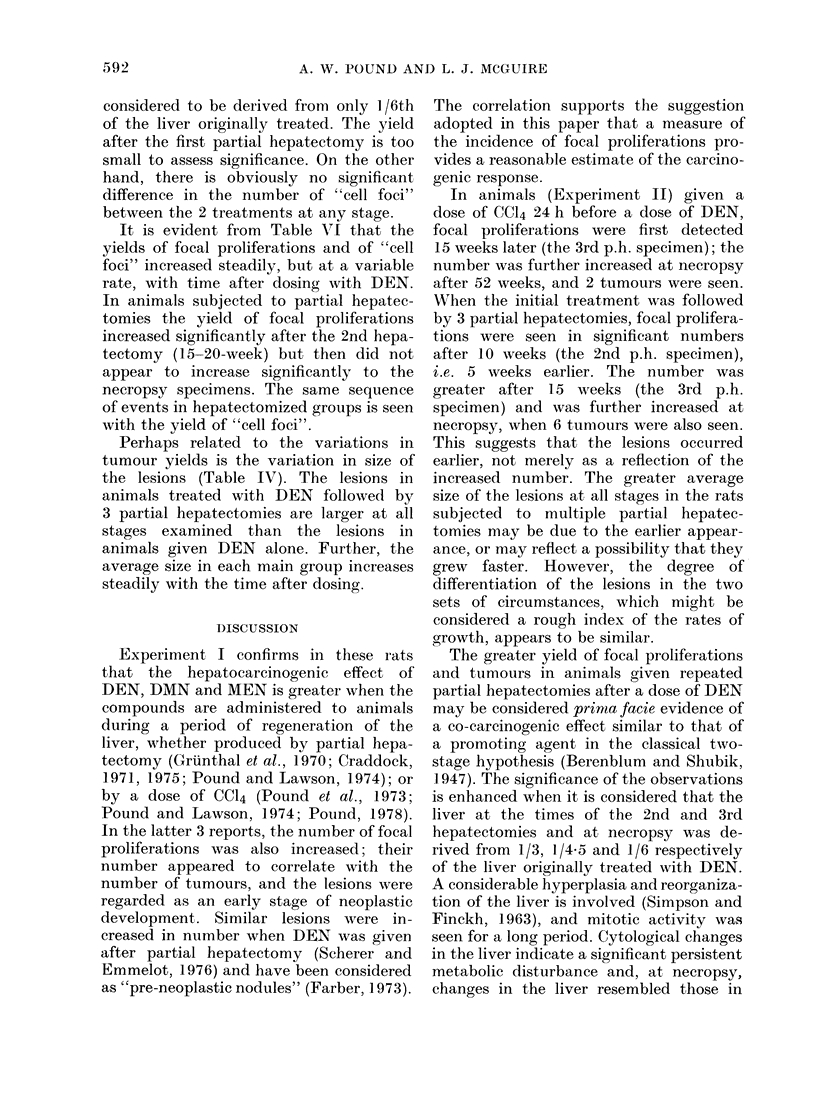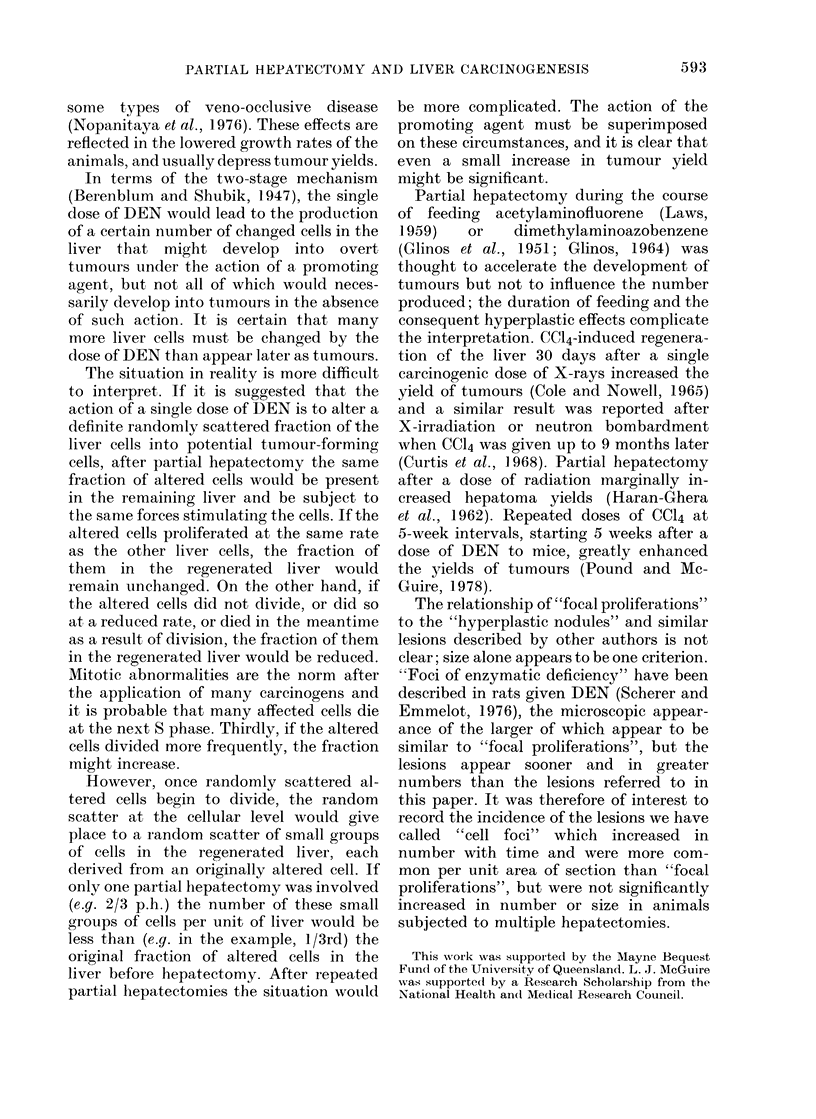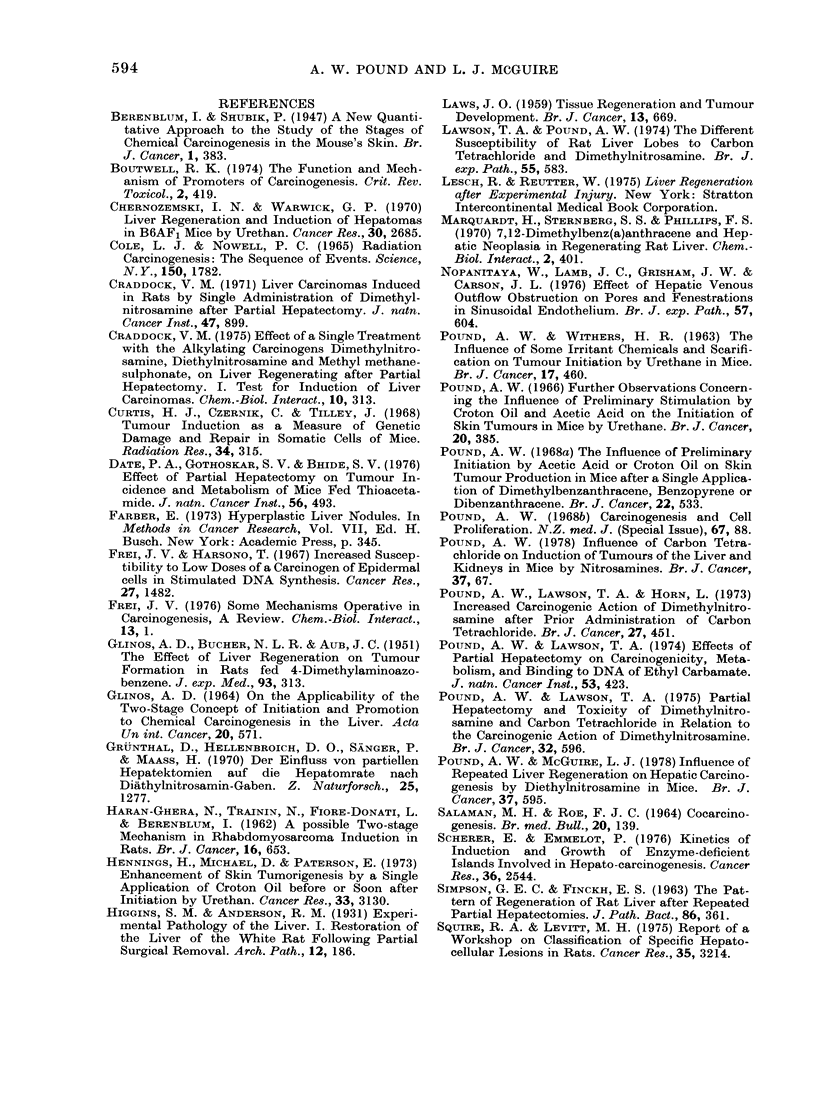# Repeated partial hepatectomy as a promoting stimulus for carcinogenic response of liver to nitrosamines in rats.

**DOI:** 10.1038/bjc.1978.88

**Published:** 1978-04

**Authors:** A. W. Pound, L. J. McGuire

## Abstract

**Images:**


*Br. J. Cancer* (1978) **37**, 585

### REPEATED PARTIAL HEPATECTOMY AS A PROMOTING STIMULUS FOR CARCINOGENIC RESPONSE OF LIVER TO NITROSAMINES IN RATS

A. W. POUND AND L. J. McGUIRE

*From the Department of Pathology, University of Queensland, Brisbane, Australia*

Received 14 November 1977 Accepted 28 December 1977

**Summary.—Partial hepatectomy 24 h *before* a single i.p. dose of dimethylnitrosamine, diethylnitrosamine or ethylmethylnitrosamine increased the carcinogenic response in the liver of rats as determined by the number of tumours and the number of "focal proliferations" produced.**

**Secondly, in rats given a single i.p. dose of diethylnitrosamine, 3 partial hepatectomies 5, 10 and 15 weeks *after* dosing the animals increased the carcinogenic response in the liver. The stimulus of repeated partial hepatectomy therefore appears to act as a "promoting agent" for liver carcinogenesis, that is if the single dose of diethylnitrosamine is regarded as an "initiating agent" in the terms of the two-stage hypothesis.**

MANY tissues become more susceptible to a carcinogen when they are induced to proliferate. Thus the number of tumours in the skin is increased when the cells are stimulated to proliferate by chemical or physical means before the administration of urethane (Pound and Withers, 1963; Pound, 1966) or 9:10-dimethylbenz(a)-anthracene (Pound, 1968*a*; Frei and Harsono, 1967). The liver proliferating after partial hepatectomy is more susceptible to the carcinogenic action of ethyl carbamate (Pound, 1968*b*; Chernozemski and Warwick, 1970; Pound and Lawson, 1974), dimethylbenz(a)anthracene (Pound, 1968*b*; Marquardt *et al.*, 1970), dimethylnitrosamine (Craddock, 1975; Pound and Lawson, 1975), diethylnitrosamine (Grünthal *et al.*, 1970), thioacetamide (Date *et al.*, 1976) and other agents. The administration of nitrosamines during the regeneration phase after a hepatonecrotic dose of $CCl_4$ also results in an increased tumour yield (Pound *et al.*, 1973; Pound and Lawson, 1975; Pound, 1978). Most data suggest that the effect is greatest when cell proliferation is most active. It has not been possible to ascribe the increased susceptibility to a precisely defined phase of the cell cycle (Hennings *et al.*, 1973; Chernozemski and Warwick, 1970; Pound and Lawson, 1974).

In addition to overt tumours, lesions referred to as "focal proliferations" (Pound *et al.*, 1973, Pound, 1978) occurred in the liver. These also increased in number in animals given nitrosamine after a dose of $CCl_4$. They were regarded as early stages of tumour development, and appeared to be similar to certain lesions found in livers subjected to other carcinogens, *e.g.* hyperplastic nodules, pre-neoplastic nodules (Farber, 1973) or foci of cellular proliferation (Squire and Levitt, 1975).

Several chemicals that are carcinogenic for skin, when administered in a dosage that leads to few or no tumours, nonetheless produce a more or less permanent change in the cells, referred to as "initiation", such that later repeated applications of a "promoting" agent, that itself is noncarcinogenic or very weakly so (*e.g.* croton oil) leads to the production of significant numbers of tumours. These observations form the basis of the "two-stage hypothesis" (Berenblum and Shubik, 1947; Salaman and Roe, 1964). The mechanism of promotion is not known, but stimulation of cell division, caused by all known promoters, may be a significant factor (Boutwell, 1974; Frei, 1976). The two-stage hypothesis has been developed mainly in relation to carcinogenesis in the skin, but has been invoked to explain some features of carcinogenesis in other tissues.

Partial hepatectomy in rats is followed by a phase of cell proliferation during which the liver mass is reconstituted in about 7 days (reviewed in Lesch and Reutter, 1975). Even after 5 partial hepatectomies the original liver mass is substantially regained, with little disturbance of the histological structure (Simpson and Finckh, 1963). This paper confirms the effect of partial hepatectomy *before* administration of some carcinogenic nitrosamines in rats, and tests the hypothesis that repeated partial hepatectomies *after* a single dose of diethylnitrosamine would enhance the carcinogenic response in the liver.

### MATERIALS AND METHODS

*Animals.*—Rats were random-bred Sprague -Dawley males from the Central Animal Breeding House, University of Queensland. They were about 7 weeks of age and 180±20 g in weight in Experiment I; and about 12 weeks old and 270±25 g in weight for Experiment II. The animals were maintained on a high (20%) protein diet with an adequate vitamin and mineral supplement (obtained as pellets from Bunge Ltd., Australia). The diet and water were freely available.

*Chemicals.*—Diethylnitrosamine (purest grade) DEN, was obtained from Fluka AG. Chemische Fabricke, Switzerland. Dimethylnitrosamine (purest grade) DMN, was obtained from Merck-Schuchardt, Federal Republic of West Germany. Ethylmethylnitrosamine (MEN) was synthesized (Pound, 1978). Carbon tetrachloride A.R., $CCl_4$, was obtained from Ajax Chemical Co., Auburn, N.S.W. Pentobarbitone sodium (Nembutal) was from Abbott Laboratories Pty. Ltd., Sydney, N.S.W., as a solution containing 60 mg/ml. Nitrosamines were administered by i.p. injection of 0·2 ml of a solution in 0·9% saline. $CCl_4$ was administered in 1 ml olive oil by gastric intubation.

*Partial hepatectomy.*—For the single 2/3 partial hepatectomy (p.h.) of Experiment I ether anaesthesia was used to avoid the effects of barbiturates on microsomal enzymes involved in nitrosamine metabolism. For repeated p.h. of Experiment II the anaesthetic was Nembutal, 0·1 ml/100 g, i.p. The technique of Higgins and Anderson (1931) was followed, but only a single ligature was used, to avoid local tissue reactions. Repeated p.h. were hindered by adhesions. Infection as such was not a major problem. A stitch abscess was seldom seen during later operations, and was found in only one rat at necropsy. The 1st p.h. consisted of removal of the median and left lateral lobes, which removed 2/3 of the liver. The 2nd p.h. consisted of removal of the right caudate lobe, which constituted about 1/3 of the now regenerated liver. The 3rd p.h. consisted of removal of 1/2 of the right lateral lobe, which removed about 1/4 of the regenerated liver. The 3rd operation was extremely difficult because of adhesions, the fragility of the liver and the site of the inferior vena cava, which traverses the lobe and tethers it to the diaphragm; in consequence there was a significant mortality.

*Experiment I.*—Three lots of 32 rats, randomly constituted, were divided into 2 equal groups, a and b. Group a animals were subjected to 2/3 p.h. and dosed with 100 mg DEN/kg, 20 mg DMN/kg or 30 mg MEN/kg respectively to each lot, 24 h later. Group b animals were subjected to laparotomy and handling of the liver only, and given the same doses of nitrosamines respectively to each lot, at the same time as the animals of Group a. The treatments on any day were randomized, and were carried out over a period of 6 days.

*Experiment II.*—Eighty rats were given a hepatonecrotic dose of 1·0 ml $CCl_4$/kg followed after 24 h by a dose of 100 mg DEN/kg. Five to 7 weeks later the animals were given the 1st p.h., followed about 5 weeks later by the 2nd, and, a further 5 weeks later, by the 3rd. Because of the numbers involved, the intervals between p.h. could not be maintained accurately. One group of 50 rats received the same dose of $CCl_4$ followed by the same dose of DEN 24 h later and no further treatment. A control group of 50 rats had the same schedule of repeated p.h. The portion of liver removed at each p.h. was examined histologically, and at the same time randomly selected rats from the other group were killed for histological examination of the livers.

Animals that died have not been considered, since the majority were postoperative.

The surviving animals were killed after 12–15 months in Experiment I and after 12 months in Experiment II, and the livers and kidneys examined by cutting into slices. Random slices of each liver and full sections of both kidneys were taken for section. Macroscopically visible tumours were counted, their mean diameters determined, and processed independently for histological sections.

*Histological methods*.—Specimens were fixed in 10% formol-saline, phosphate-buffered at pH 7·2, dehydrated in alcohols and embedded in paraffin in the usual way. Sections cut at 5 $\mu$m were stained routinely with haematoxylin and eosin (HE). PAS stain was used to identify glycogen.

Sections of livers were systematically scanned for the presence of "focal proliferations" and "cell foci" (see below), the numbers of which were counted. Mean diameters of the lesions were measured with an eyepiece micrometer. The area scanned was derived by outlining the section on millimetre-square graph paper. In order to obtain an adequate area, multiple sections through some blocks were necessary; these were separated by 0·5–1 mm to avoid counting lesions more than once. Measurements and counts were repeated 3 times, with similar results.

### RESULTS

The characteristics of the hepatocellular lesions in nitrosamine-treated animals were the same in the two experiments. The lesions in the rat have been described (Pound *et al.*, 1973). In summary, hepatocellular tumours were creamy-white nodules that were counted and measured by naked eye. Three types were recognized according to the character of the cells comprising them: clear-cell, liver cell and dark cell, and there was evidence of expansive and invasive growth. Mitoses were not uncommon, but there were only minor degrees of cell pleomorphism.

"Focal proliferations" consisted of small lesions seen on scanning sections of the liver microscopically. When presenting on the surface, they could occasionally be seen as small white spots. The lesions consisted of groups of cells characterized by expansive growth and invasion of the surrounding liver, sometimes into veins. The cells were usually of uniform type with nuclei of uniform size which, however, often differed from those in the surrounding hepatocytes where nuclear size was more variable. These lesions also fell into 3 types (clear-cell type (Fig. 1), liver-cell type (Fig. 2) or dark-cell type (Fig. 3)) according to the cells comprising them, clearly analogous to the types of hepatocellular tumours.

In addition to the above lesions, small foci of uniform liver cells differing in cytological characteristics and nuclear size from normal hepatocytes were commonly seen. These were a clear-cell type (Fig. 4) and a dark-cell type, resembling the cells in the types of tumour and focal proliferations. A liver-cell lesion of this type would

FIG. 1.—Focal proliferation of clear-cell type, 0·7 mm diam., showing cell characteristics and expansive growth compressing surrounding liver. HE ×150.

FIG. 2.—Focal proliferation of liver-cell type, 1 mm diam., showing cell characteristics, a common trabecular arrangement of the cells and expansive growth compressing the surrounding liver. HE ×150.

FIG. 3.—Focal proliferation of dark-cell type, 0·25 mm diam., showing cell characteristics and invasive type of growth into the surrounding liver. HE ×230.

not be detectable with any conviction. These lesions did not show evidence of active expansive growth and were often ill-defined. It was of interest to examine the nature of these lesions, and they have been referred to below as "cell foci".

*Experiment I*

The number of deaths in the animals of Experiment I was small, and obviously no statistically significant difference exists between the groups (Table 1). At necropsy, the livers appeared normal, apart from occasional tumours. Histologically the sinusoids in the central zones of the lobules were a little dilated but there was no fibrosis or cirrhosis. One animal had a reticulum-cell sarcoma of the liver and spleen.

*Hepatocellular tumours and related lesions.*—The distribution of hepatocellular tumours and focal proliferations is shown in Table I. Tumours were small in number and were seen only in animals that had partial hepatectomy before dosing with the nitrosamines, of which DEN was the most potent. The number of focal proliferations with each nitrosamine is also greater in the group that had a previous partial hepatectomy, but the increase is statistically significant only in the case of DEN ($\chi^2 = 19{\cdot}9$, 1 d.f., $P < 0{\cdot}001$). However, the data with DMN and MEN support previous data that the proliferating liver is the more susceptible. Kidney tumours appeared only in animals that had been given partial hepatectomy before dosing.

FIG. 4.—Cell focus of clear-cell type, 0·2 mm diam., showing cell characteristics similar to those of this type of focal proliferation and tumour. In this example there is some evidence of expansive growth, but often this is absent. HE ×230.

*Experiment II*

In Experiment II a substantial number of animals died during or soon after procedures of multiple hepatectomy. No tumours were noted at operations, and at these times the livers did not appear abnormal. Nor did the incidence or character of lesions seen on screening a random sample of livers from animals that died appear to differ from those in the pieces of liver removed in animals of the same group, but the lesions were not counted. For these reasons, any influence of mortality on the assessment of results has been ignored.

Histologically, the portions of liver resected showed no disturbance of the liver architecture. The liver sinusoids were often less prominent than normal, because of enlargement of the hepatocytes associated with the presence of glycogen. This change was greater in DEN-treated animals and suggests a functional disturbance of the liver in the periods between hepatectomies. The number of cells in mitosis in these DEN-treated groups was greater than in normal animals by a factor of at least 2 or 3, although formal counts were not made. There were numerous cells with large polyploid nuclei.

*Necropsy findings*.—The weights of the

TABLE I.—*Incidence of Hepatocellular Lesions and Kidney Tumours in Rats subjected to 2/3rd Partial Hepatectomy (p.h.) 24 h before i.p. Administration of Diethylnitrosamine (DEN), Dimethylnitrosamine (DMN) or Ethylmethylnitrosamine (MEN). Experiment I.*

| | | | Rats with liver tumours | | | | | | |
|---|---|---|---|---|---|---|---|---|---|
| | | | Hepatocellular tumours | | Focal proliferations† | | Rats with kidney tumours | | |
| Nitrosamine dose | Prior p.h. | Surviving rats* | No. of rats | No. of lesions | No. of rats | No. of lesions | No. of rats | Clear-cell adenomas | Mesenchymal tumours |
| DEN | + | 14 | 4 | 5‡ | 12 | 44 | 5 | 8§ | 0 |
| 100 mg/kg | — | 16 | 0 | 0 | 7 | 14 | 0 | 0 | 0 |
| DMN | + | 15 | 2 | 2¶ | 9 | 13 | 3 | 2 | 2 |
| 20 mg/kg | — | 14 | 0 | 0 | 4 | 7 | 0 | 0 | 0 |
| MEN | + | 16 | 1 | 1 | 9 | 11 | 2 | 2 | 0 |
| 30 mg/kg | — | 16 | 0 | 0 | 3 | 5 | 0 | 0 | 0 |

* out of 16.
† Area of liver scanned about 3 $cm^2$ per rat.
‡ Lesions from 2 to 12 mm diameter.
§ 6 small lesions 0·5–1 mm diameter (4 in one mouse), 2 tumours >1 cm.
¶ Two lesions 1 and 3 mm diameter.

TABLE II.—*Weights (g) of Animals and Livers at Necropsy Experiment II*

| Treatment | Animals | Livers | Liver as % body wt |
|---|---|---|---|
| * DEN+Multiple p.h. | 290±35 | 9·9±1·3 | 3·41±0·34 |
| * DEN | 309±37 | 12·2±1·7 | 3·93±0·33 |
| Multiple p.h. | 350±47 | 10·7±1·3 | 3·06±0·32 |
| † Normal rats | 434±44 | 14·3±1·9 | 3·30±0·29 |

* Animals received a hepatonecrotic dose of $CCl_4$ 24 h before 100 mg/DEN/kg.

† 12 months of age.

animals and their livers at necropsy, Table II, show that multiple hepatectomies retarded the growth of the animals, and that the retardation was greater in animals that had been dosed with DEN. The weight of the liver was regained to the extent of 93% in partially hepatectomized animals; but, interestingly, in animals given DEN the livers, although of less weight than in normal adult animals, constituted a higher percentage of the body weight.

The livers of control animals were within normal limits macroscopically and microscopically, apart from the altered configurations consequent upon the multiple partial hepatectomies. Livers of DEN-treated rats also appeared normal, apart from slightly depressed brownish-red patches on the surface which were larger in the group subjected to repeated partial hepatectomy. The histological architecture of these livers was normal, but the sinusoids were dilated and the liver trabeculae narrowed in the central zones of the lobules. In these zones glycogen staining was depleted. The sinusoidal dilatation was of greater extent in the hepatectomized group and, where the areas impinged on the surface, they accounted for the brownish red patches referred to above. There was no hepatic fibrosis or cirrhosis.

No hepatocellular or other lesions were seen on scanning sections of the livers of the control animals. In the DEN-treated animals, small areas of bile-duct proliferation were occasionally seen, similar to those produced by DMN and previously reported (Pound *et al.*, 1973). These lesions and the control rats are not considered further.

*Hepatocellular tumours and related lesions.*—The number of tumours, focal proliferations and "cell foci" found in the animals treated with DEN 24 h after a dose of $CCl_4$, and other relevant data are shown in Table III. The distribution of sizes of the lesions is set out in Table IV.

The number of tumours in the surviving rats is greater in the animals subjected to 3 partial hepatectomies, but not significantly so ($\chi^2$ corrected = 1·79; N.S.); the numbers are too small for a realistic evaluation. The fact that the liver at this stage is derived from only a fraction of the liver originally treated with DEN remains for discussion.

The yields of focal proliferations and "cell foci" cannot be compared on this basis, because of variation in areas of liver scanned. These areas were obtained from different lobes in the liver, which may respond differently (Lawson and Pound, 1974). It has to be assumed that the resected lobes are representative of the whole liver, and that the number of lesions counted per unit area provides a measure of the number of lesions per unit volume of liver which can be used as a basis for comparison, since a few random sections are likely to intersect only a fraction of the lesions present.

Statistical data on comparisons based on these assumptions for the raw results are set out in Tables V and VI. The data of Table V suggest that the incidence of focal proliferations is significantly greater in the animals subjected to partial hepatectomies after DEN after the 2nd partial hepatectomy (15-week) but that the increase found in the necropsy specimens is not statistically significant, ignoring the fact that the liver at this stage may be

TABLE III.—*Numbers of Tumours, Focal Proliferations and Cell Foci found in Rats treated with DEN, and DEN followed by Multiple Partial Hepatectomies (p.h.). Experiment II.*

| Treatment† | Group | No. of p.h. | Time after dosing | No. of rats | Area of liver scanned ($cm^2$) | Number of "cell foci" | Number of focal prolifera-tions (FP) | No. of rats with FP | Hepato-cellular tumours |
|---|---|---|---|---|---|---|---|---|---|
| DEN+ | R1/2 | 1 | *10 weeks | 14 | 29·2 | 28 | 5 | 4 | 0 |
| Multiple p.h. | R1/3 | 2 | *15 weeks | 14 | 49·6 | 84 | 61 | 10 | 0 |
| | R1/4 | 3 | 52 weeks | 18 | 60·1 | 117 | 72 | 16 | 6 |
| | R2/2 | | 10 weeks | 10 | 32·0 | 26 | 0 | 0 | 0 |
| DEN | R2/3 | | 15 weeks | 10 | 52·2 | 76 | 20 | 6 | 0 |
| | R2/4 | | 52 weeks | 17 | 54·4 | 105 | 49 | 15 | 2 |

* Specimens taken at 2nd and 3rd p.h. respectively.
† Animals treated with a hepatonecrotic dose of $CCl_4$ 24 h before DEN.

TABLE IV.—*Distribution of Size of Lesions in Rats treated with DEN, and DEN followed by Repeated p.h., at Different Times after Treatment*

| Treatment group | No. of focal proliferations with diameter (mm) | | | | | | Focal proliferations mean diam. (mm) (range) | Cell foci. mean diam. (mm) (range) | Tumour diameters (mm) |
|---|---|---|---|---|---|---|---|---|---|
| | 0·22 to 0·43 | 0·44 to 0·65 | 0·66 to 0·87 | 0·88 to 1·09 | 1·10 to 1·31 | 1·32 to 1·53 | | | |
| R1/2 (10 wks) | 6 | — | — | — | — | — | 0·31 (0·13–0·41) | 0·11 (0·06–0·24) | — |
| R1/3 (15 wks) | 37 | 8 | 10 | 6 | — | — | 0·44 (0·19–1·18) | 0·14 (0·04–0·29) | — |
| R1/4 (52 wks) | 22 | 27 | 15 | 4 | 3 | 1 | 0·65 (0·22–1·65) | 0·16 (0·06–0·27) | 2, 3, 3 2, 4, 4 |
| R2/2 (10 wks) | — | — | — | — | — | — | — | 0·13 (0·06–0·24) | — |
| R2/3 (15 wks) | 20 | — | — | — | — | — | 0·26 (0·09–0·33) | 0·11 (0·06–0·24) | — |
| R2/4 (52 wks) | 28 | 18 | 1 | 1 | 1 | — | 0·39 (0·22–1·10) | 0·21 (0·06–0·24) | 4, 2 |

TABLE V.—*Statistical Analysis of the Effect of Repeated Partial Hepatectomies (p.h.) on Numbers of Cell Foci and Focal Proliferations at Different Times after Dosing with DEN*

| | | | |
|---|---|---|---|
| Cell foci | 10 weeks | p.h. *vs* no p.h. | $\chi^2 = 0{\cdot}04$; N.S. |
| | 15 weeks | p.h. *vs* no p.h. | $\chi^2 = 1{\cdot}0$; N.S. |
| | 52 weeks | p.h. *vs* no p.h. | $\chi^2 = 0{\cdot}04$; N.S. |
| Focal proliferations | 10 weeks | p.h. *vs* no p.h. | $\chi^2 = 2{\cdot}3$; N.S. |
| | 15 weeks | p.h. *vs* no p.h. | $\chi^2 = 22{\cdot}8$; $P < 0{\cdot}001$ |
| | 52 weeks | p.h. *vs* no p.h. | $\chi^2 = 2{\cdot}38$; N.S. |

1 d.f. in each case

TABLE VI.—*Statistical Analysis of the Increase in Numbers of Cell Foci and Focal Proliferation with Time after Dosing and the Effect of Repeated Partial Hepatectomies (p.h.)*

| | | 10 weeks *vs* 15 weeks | 15 weeks *vs* necropsy |
|---|---|---|---|
| Cell foci | p.h. | $\chi^2 = 6{\cdot}98$; $P < 0{\cdot}01$ | $\chi^2 = 0{\cdot}95$; N.S. |
| | no p.h. | $\chi^2 = 6{\cdot}88$; $P < 0{\cdot}01$ | $\chi^2 = 3{\cdot}53$; $0{\cdot}1 > P > 0{\cdot}05$ |
| Focal proliferations | p.h. | $\chi^2 = 24{\cdot}1$; $P < 0{\cdot}001$ | $\chi^2 = 0{\cdot}02$; N.S. |
| | no p.h. | $\chi^2 = 9{\cdot}85$; $P < 0{\cdot}005$ | $\chi^2 = 11{\cdot}1$; $P < 0{\cdot}001$ |

1 d.f. in each case considered to be derived from only 1/6th of the liver originally treated. The yield after the first partial hepatectomy is too small to assess significance. On the other hand, there is obviously no significant difference in the number of "cell foci" between the 2 treatments at any stage.

It is evident from Table VI that the yields of focal proliferations and of "cell foci" increased steadily, but at a variable rate, with time after dosing with DEN. In animals subjected to partial hepatectomies the yield of focal proliferations increased significantly after the 2nd hepatectomy (15–20-week) but then did not appear to increase significantly to the necropsy specimens. The same sequence of events in hepatectomized groups is seen with the yield of "cell foci".

Perhaps related to the variations in tumour yields is the variation in size of the lesions (Table IV). The lesions in animals treated with DEN followed by 3 partial hepatectomies are larger at all stages examined than the lesions in animals given DEN alone. Further, the average size in each main group increases steadily with the time after dosing.

### DISCUSSION

Experiment I confirms in these rats that the hepatocarcinogenic effect of DEN, DMN and MEN is greater when the compounds are administered to animals during a period of regeneration of the liver, whether produced by partial hepatectomy (Grünthal *et al.*, 1970; Craddock, 1971, 1975; Pound and Lawson, 1974); or by a dose of $CCl_4$ (Pound *et al.*, 1973; Pound and Lawson, 1974; Pound, 1978). In the latter 3 reports, the number of focal proliferations was also increased; their number appeared to correlate with the number of tumours, and the lesions were regarded as an early stage of neoplastic development. Similar lesions were increased in number when DEN was given after partial hepatectomy (Scherer and Emmelot, 1976) and have been considered as "pre-neoplastic nodules" (Farber, 1973). The correlation supports the suggestion adopted in this paper that a measure of the incidence of focal proliferations provides a reasonable estimate of the carcinogenic response.

In animals (Experiment II) given a dose of $CCl_4$ 24 h before a dose of DEN, focal proliferations were first detected 15 weeks later (the 3rd p.h. specimen); the number was further increased at necropsy after 52 weeks, and 2 tumours were seen. When the initial treatment was followed by 3 partial hepatectomies, focal proliferations were seen in significant numbers after 10 weeks (the 2nd p.h. specimen), *i.e.* 5 weeks earlier. The number was greater after 15 weeks (the 3rd p.h. specimen) and was further increased at necropsy, when 6 tumours were also seen. This suggests that the lesions occurred earlier, not merely as a reflection of the increased number. The greater average size of the lesions at all stages in the rats subjected to multiple partial hepatectomies may be due to the earlier appearance, or may reflect a possibility that they grew faster. However, the degree of differentiation of the lesions in the two sets of circumstances, which might be considered a rough index of the rates of growth, appears to be similar.

The greater yield of focal proliferations and tumours in animals given repeated partial hepatectomies after a dose of DEN may be considered *prima facie* evidence of a co-carcinogenic effect similar to that of a promoting agent in the classical two-stage hypothesis (Berenblum and Shubik, 1947). The significance of the observations is enhanced when it is considered that the liver at the times of the 2nd and 3rd hepatectomies and at necropsy was derived from 1/3, 1/4·5 and 1/6 respectively of the liver originally treated with DEN. A considerable hyperplasia and reorganization of the liver is involved (Simpson and Finckh, 1963), and mitotic activity was seen for a long period. Cytological changes in the liver indicate a significant persistent metabolic disturbance and, at necropsy, changes in the liver resembled those in some types of veno-occlusive disease (Nopanitaya *et al.*, 1976). These effects are reflected in the lowered growth rates of the animals, and usually depress tumour yields.

In terms of the two-stage mechanism (Berenblum and Shubik, 1947), the single dose of DEN would lead to the production of a certain number of changed cells in the liver that might develop into overt tumours under the action of a promoting agent, but not all of which would necessarily develop into tumours in the absence of such action. It is certain that many more liver cells must be changed by the dose of DEN than appear later as tumours.

The situation in reality is more difficult to interpret. If it is suggested that the action of a single dose of DEN is to alter a definite randomly scattered fraction of the liver cells into potential tumour-forming cells, after partial hepatectomy the same fraction of altered cells would be present in the remaining liver and be subject to the same forces stimulating the cells. If the altered cells proliferated at the same rate as the other liver cells, the fraction of them in the regenerated liver would remain unchanged. On the other hand, if the altered cells did not divide, or did so at a reduced rate, or died in the meantime as a result of division, the fraction of them in the regenerated liver would be reduced. Mitotic abnormalities are the norm after the application of many carcinogens and it is probable that many affected cells die at the next S phase. Thirdly, if the altered cells divided more frequently, the fraction might increase.

However, once randomly scattered altered cells begin to divide, the random scatter at the cellular level would give place to a random scatter of small groups of cells in the regenerated liver, each derived from an originally altered cell. If only one partial hepatectomy was involved (*e.g.* 2/3 p.h.) the number of these small groups of cells per unit of liver would be less than (*e.g.* in the example, 1/3rd) the original fraction of altered cells in the liver before hepatectomy. After repeated partial hepatectomies the situation would be more complicated. The action of the promoting agent must be superimposed on these circumstances, and it is clear that even a small increase in tumour yield might be significant.

Partial hepatectomy during the course of feeding acetylaminofluorene (Laws, 1959) or dimethylaminoazobenzene (Glinos *et al.*, 1951; Glinos, 1964) was thought to accelerate the development of tumours but not to influence the number produced; the duration of feeding and the consequent hyperplastic effects complicate the interpretation. $CCl_4$-induced regeneration of the liver 30 days after a single carcinogenic dose of X-rays increased the yield of tumours (Cole and Nowell, 1965) and a similar result was reported after X-irradiation or neutron bombardment when $CCl_4$ was given up to 9 months later (Curtis *et al.*, 1968). Partial hepatectomy after a dose of radiation marginally increased hepatoma yields (Haran-Ghera *et al.*, 1962). Repeated doses of $CCl_4$ at 5-week intervals, starting 5 weeks after a dose of DEN to mice, greatly enhanced the yields of tumours (Pound and McGuire, 1978).

The relationship of "focal proliferations" to the "hyperplastic nodules" and similar lesions described by other authors is not clear; size alone appears to be one criterion. "Foci of enzymatic deficiency" have been described in rats given DEN (Scherer and Emmelot, 1976), the microscopic appearance of the larger of which appear to be similar to "focal proliferations", but the lesions appear sooner and in greater numbers than the lesions referred to in this paper. It was therefore of interest to record the incidence of the lesions we have called "cell foci" which increased in number with time and were more common per unit area of section than "focal proliferations", but were not significantly increased in number or size in animals subjected to multiple hepatectomies.

This work was supported by the Mayne Bequest Fund of the University of Queensland. L. J. McGuire was supported by a Research Scholarship from the National Health and Medical Research Council.

REFERENCES

BERENBLUM, I. & SHUBIK, P. (1947) A New Quantitative Approach to the Study of the Stages of Chemical Carcinogenesis in the Mouse's Skin. *Br. J. Cancer*, **1**, 383.

BOUTWELL, R. K. (1974) The Function and Mechanism of Promoters of Carcinogenesis. *Crit. Rev. Toxicol.*, **2**, 419.

CHERNOZEMSKI, I. N. & WARWICK, G. P. (1970) Liver Regeneration and Induction of Hepatomas in $B6AF_1$ Mice by Urethan. *Cancer Res.*, **30**, 2685.

COLE, L. J. & NOWELL, P. C. (1965) Radiation Carcinogenesis: The Sequence of Events. *Science, N.Y.*, **150**, 1782.

CRADDOCK, V. M. (1971) Liver Carcinomas Induced in Rats by Single Administration of Dimethylnitrosamine after Partial Hepatectomy. *J. natn. Cancer Inst.*, **47**, 899.

CRADDOCK, V. M. (1975) Effect of a Single Treatment with the Alkylating Carcinogens Dimethylnitrosamine, Diethylnitrosamine and Methyl methanesulphonate, on Liver Regenerating after Partial Hepatectomy. I. Test for Induction of Liver Carcinomas. *Chem.-Biol. Interact.*, **10**, 313.

CURTIS, H. J., CZERNIK, C. & TILLEY, J. (1968) Tumour Induction as a Measure of Genetic Damage and Repair in Somatic Cells of Mice. *Radiation Res.*, **34**, 315.

DATE, P. A., GOTHOSKAR, S. V. & BHIDE, S. V. (1976) Effect of Partial Hepatectomy on Tumour Incidence and Metabolism of Mice Fed Thioacetamide. *J. natn. Cancer Inst.*, **56**, 493.

FARBER, E. (1973) Hyperplastic Liver Nodules. In *Methods in Cancer Research*, Vol. VII, Ed. H. Busch. New York: Academic Press, p. 345.

FREI, J. V. & HARSONO, T. (1967) Increased Susceptibility to Low Doses of a Carcinogen of Epidermal cells in Stimulated DNA Synthesis. *Cancer Res.*, **27**, 1482.

FREI, J. V. (1976) Some Mechanisms Operative in Carcinogenesis, A Review. *Chem.-Biol. Interact.*, **13**, 1.

GLINOS, A. D., BUCHER, N. L. R. & AUB, J. C. (1951) The Effect of Liver Regeneration on Tumour Formation in Rats fed 4-Dimethylaminoazobenzene. *J. exp. Med.*, **93**, 313.

GLINOS, A. D. (1964) On the Applicability of the Two-Stage Concept of Initiation and Promotion to Chemical Carcinogenesis in the Liver. *Acta Un int. Cancer*, **20**, 571.

GRÜNTHAL, D., HELLENBROICH, D. O., SÄNGER, P. & MAASS, H. (1970) Der Einfluss von partiellen Hepatektomien auf die Hepatomrate nach Diäthylnitrosamin-Gaben. *Z. Naturforsch.*, **25**, 1277.

HARAN-GHERA, N., TRAININ, N., FIORE-DONATI, L. & BERENBLUM, I. (1962) A possible Two-stage Mechanism in Rhabdomyosarcoma Induction in Rats. *Br. J. Cancer*, **16**, 653.

HENNINGS, H., MICHAEL, D. & PATERSON, E. (1973) Enhancement of Skin Tumorigenesis by a Single Application of Croton Oil before or Soon after Initiation by Urethan. *Cancer Res.*, **33**, 3130.

HIGGINS, S. M. & ANDERSON, R. M. (1931) Experimental Pathology of the Liver. I. Restoration of the Liver of the White Rat Following Partial Surgical Removal. *Arch. Path.*, **12**, 186.

LAWS, J. O. (1959) Tissue Regeneration and Tumour Development. *Br. J. Cancer*, **13**, 669.

LAWSON, T. A. & POUND, A. W. (1974) The Different Susceptibility of Rat Liver Lobes to Carbon Tetrachloride and Dimethylnitrosamine. *Br. J. exp. Path.*, **55**, 583.

LESCH, R. & REUTTER, W. (1975) *Liver Regeneration after Experimental Injury*. New York: Stratton Intercontinental Medical Book Corporation.

MARQUARDT, H., STERNBERG, S. S. & PHILLIPS, F. S. (1970) 7,12-Dimethylbenz(a)anthracene and Hepatic Neoplasia in Regenerating Rat Liver. *Chem.-Biol. Interact.*, **2**, 401.

NOPANITAYA, W., LAMB, J. C., GRISHAM, J. W. & CARSON, J. L. (1976) Effect of Hepatic Venous Outflow Obstruction on Pores and Fenestrations in Sinusoidal Endothelium. *Br. J. exp. Path.*, **57**, 604.

POUND, A. W. & WITHERS, H. R. (1963) The Influence of Some Irritant Chemicals and Scarification on Tumour Initiation by Urethane in Mice. *Br. J. Cancer*, **17**, 460.

POUND, A. W. (1966) Further Observations Concerning the Influence of Preliminary Stimulation by Croton Oil and Acetic Acid on the Initiation of Skin Tumours in Mice by Urethane. *Br. J. Cancer*, **20**, 385.

POUND, A. W. (1968*a*) The Influence of Preliminary Initiation by Acetic Acid or Croton Oil on Skin Tumour Production in Mice after a Single Application of Dimethylbenzanthracene, Benzopyrene or Dibenzanthracene. *Br. J. Cancer*, **22**, 533.

POUND, A. W. (1968*b*) Carcinogenesis and Cell Proliferation. *N.Z. med. J.* (Special Issue), **67**, 88.

POUND, A. W. (1978) Influence of Carbon Tetrachloride on Induction of Tumours of the Liver and Kidneys in Mice by Nitrosamines. *Br. J. Cancer*, **37**, 67.

POUND, A. W., LAWSON, T. A. & HORN, L. (1973) Increased Carcinogenic Action of Dimethylnitrosamine after Prior Administration of Carbon Tetrachloride. *Br. J. Cancer*, **27**, 451.

POUND, A. W. & LAWSON, T. A. (1974) Effects of Partial Hepatectomy on Carcinogenicity, Metabolism, and Binding to DNA of Ethyl Carbamate. *J. natn. Cancer Inst.*, **53**, 423.

POUND, A. W. & LAWSON, T. A. (1975) Partial Hepatectomy and Toxicity of Dimethylnitrosamine and Carbon Tetrachloride in Relation to the Carcinogenic Action of Dimethylnitrosamine. *Br. J. Cancer*, **32**, 596.

POUND, A. W. & MCGUIRE, L. J. (1978) Influence of Repeated Liver Regeneration on Hepatic Carcinogenesis by Diethylnitrosamine in Mice. *Br. J. Cancer*, **37**, 595.

SALAMAN, M. H. & ROE, F. J. C. (1964) Cocarcinogenesis. *Br. med. Bull.*, **20**, 139.

SCHERER, E. & EMMELOT, P. (1976) Kinetics of Induction and Growth of Enzyme-deficient Islands Involved in Hepato-carcinogenesis. *Cancer Res.*, **36**, 2544.

SIMPSON, G. E. C. & FINCKH, E. S. (1963) The Pattern of Regeneration of Rat Liver after Repeated Partial Hepatectomies. *J. Path. Bact.*, **86**, 361.

SQUIRE, R. A. & LEVITT, M. H. (1975) Report of a Workshop on Classification of Specific Hepatocellular Lesions in Rats. *Cancer Res.*, **35**, 3214.